# Whole-body parametric [^18^F]-FDG PET/CT improves interpretation of a distant lesion as venous embolus in a lung cancer patient

**DOI:** 10.1007/s00259-020-05176-0

**Published:** 2021-01-15

**Authors:** Michael Messerli, Fotis Kotasidis, Daniela A. Ferraro, Ken Kudura, Valerie Treyer, Josephine Trinckauf, Corina Weyermann, Martin Hüllner, Philipp Kaufmann, Irene A. Burger

**Affiliations:** 1 Department of Nuclear Medicine, University Hospital Zurich, University of Zurich, Rämistrasse 100, 8091 Zürich, Switzerland; 2 GE Healthcare, Waukesha, WI USA; 3 Department of Nuclear Medicine, Cantonal Hospital of Baden, Baden, Switzerland

Despite well-known limitations of static analysis of FDG-PET images, the ease of its use leads to a widespread use of semi-quantitative indices, most commonly the standardized uptake value (SUV). In contrast to static imaging, dynamic imaging with kinetic modeling allows estimation of quantitative parameters, independent of uptake time, body weight, or injected dose, therefore potentially improving the interpretation of FDG-accumulation [1]. With improvements in scanner sensitivity and reconstruction algorithms, recently proposed multipass whole-body (WB) protocols in conjunction with image-derived input functions (IF) have made WB kinetic parameter estimation feasible [2]. Here we present a case of a patient with bronchial cancer and a venous thrombus in the left brachiocephalic vein (green arrows) with unclear FDG accumulation in the left axillary region (blue arrows) on SUV images (a). The patient was injected (134.1 MBq) and data over the heart were acquired for 7 min (blue insert: 155–180 s), followed by 10 WB passes (35 s/bed). The IF was extracted from the descending aorta and after appropriate corrections was used to estimate glucose influx (Ki) (b) and volume of distribution (Vd) (c) [3]. Note the high tumor uptake (red arrows) while missing accumulation in the axillar region on Ki (b), compared to high uptake in venous regions but low accumulation in the primary tumor on Vd (c). Standard static imaging was performed after the WB dynamic protocol (2.5 min/bed) (a), axial images are given for both areas of interest (a1/a2, scaled SUV 0-6). Only after review of the parametric images, the cause of FDG accumulation was linked to venous collaterals due to thrombosis.


